# Calcium Imaging of Basal Forebrain Activity during Innate and Learned Behaviors

**DOI:** 10.3389/fncir.2016.00036

**Published:** 2016-05-09

**Authors:** Thomas C. Harrison, Lucas Pinto, Julien R. Brock, Yang Dan

**Affiliations:** Division of Neurobiology, Department of Molecular and Cell Biology, Helen Wills Neuroscience Institute, Howard Hughes Medical Institute, University of California, BerkeleyBerkeley, CA, USA

**Keywords:** calcium imaging, mouse, basal forebrain, behavior, auditory discrimination, free moving

## Abstract

The basal forebrain (BF) plays crucial roles in arousal, attention, and memory, and its impairment is associated with a variety of cognitive deficits. The BF consists of cholinergic, GABAergic, and glutamatergic neurons. Electrical or optogenetic stimulation of BF cholinergic neurons enhances cortical processing and behavioral performance, but the natural activity of these cells during behavior is only beginning to be characterized. Even less is known about GABAergic and glutamatergic neurons. Here, we performed microendoscopic calcium imaging of BF neurons as mice engaged in spontaneous behaviors in their home cages (innate) or performed a go/no-go auditory discrimination task (learned). Cholinergic neurons were consistently excited during movement, including running and licking, but GABAergic and glutamatergic neurons exhibited diverse responses. All cell types were activated by overt punishment, either inside or outside of the discrimination task. These findings reveal functional similarities and distinctions between BF cell types during both spontaneous and task-related behaviors.

## Introduction

The basal forebrain (BF) comprises several nuclei containing cholinergic projection neurons (Mesulam et al., 1983; Semba, 2000; Jones, 2004), and it has been implicated in arousal, attention, plasticity and learning/memory (Buzsaki et al., 1988; Richardson and DeLong, 1990; Wilson and Rolls, 1990a; Everitt and Robbins, 1997; Kilgard and Merzenich, 1998; Fournier et al., 2004; Sarter et al., 2005; Herrero et al., 2008; Disney et al., 2012; Chubykin et al., 2013; Froemke et al., 2013; Minces et al., 2013; Rokem and Silver, 2013; Eggermann et al., 2014; Kang et al., 2014; Kim et al., 2015; Lin et al., 2015; Liu et al., 2015). Degeneration of cholinergic BF neurons in humans is an early event in dementia, including Alzheimer's disease (Grothe et al., 2014), and the associated symptoms are commonly treated with pharmacological manipulations of the cholinergic system (Robbins et al., 1997; Birks, 2006). In animal studies, lesions or inactivation of the cholinergic system have also been shown to impair attention and memory (Chiba et al., 1999; McGaughy et al., 2000; Pinto et al., 2013). Conversely, electrical stimulation of the BF or selective activation of BF cholinergic neurons induced arousal (Han et al., 2014; Irmak and de Lecea, 2014; Xu et al., 2015), cortical activation (Metherate and Ashe, 1993; Goard and Dan, 2009; Kalmbach et al., 2012), and an improvement in performance on a sensory discrimination task (Pinto et al., 2013).

Although much has been learned from manipulations of the cholinergic system, the natural pattern of activity of BF cholinergic neurons remains poorly understood. Electrophysiological recordings from rats (Lee et al., 2005) and mice (Xu et al., 2015) showed that these neurons are active during both wakefulness and rapid-eye-movement (REM) sleep but are silent during non-REM sleep. In addition to sleep/wake-related firing rate modulation on the order of minutes, cholinergic neurons can also respond to reward and punishment on a time scale of tens of milliseconds (Hangya et al., 2015). Furthermore, locomotion-induced enhancement of neuronal responses in the primary visual cortex has been shown to depend on cholinergic inputs from the BF (Fu et al., 2014; Lee et al., 2014), but whether BF cholinergic neurons are activated during locomotion remains unclear.

In addition to cholinergic cells, which in fact represent a small minority of BF neurons, the BF also contains glutamatergic and GABAergic neurons. All of these cell classes form reciprocal connections within the BF (Yang et al., 2014; Xu et al., 2015). Non-cholinergic BF neurons form widespread projections (Gritti et al., 2006; Henny and Jones, 2008; Zaborszky et al., 2015) and are likely to be involved in arousal and attentional modulation (Brown and McKenna, 2015; Raver and Lin, 2015). For example, a recent study showed that the activity of non-cholinergic BF neurons was correlated with sustained attention (Hangya et al., 2015), and others have found that non-cholinergic BF neurons encode motivational salience (Lin and Nicolelis, 2008; Avila and Lin, 2014). However, whether the neurons recorded in these studies are glutamatergic or GABAergic is unknown. Characterizing the behaviorally relevant signals encoded by each cell type is crucial for understanding the function of the BF circuit.

In this study, we performed microendoscopic calcium imaging of cholinergic, GABAergic, and glutamatergic neurons in the BF of mice as they engaged in spontaneous behaviors in their home cages or performed a go/no-go auditory discrimination task. We found that cholinergic neurons were consistently excited by movement (including licking and running) and responded to overt punishment regardless of behavioral context. In contrast, GABAergic and glutamatergic neurons exhibited diverse activity changes during movement and responded preferentially to punishment delivered during the go/no-go task. These results provide the first comprehensive characterization of cell-type-specific BF activity during innate and learned behaviors.

## Materials and methods

### Animals and surgery

All procedures were approved by the Animal Care and Use Committee at the University of California Berkeley. The experiments involved male and female mice aged 2–6 months of the following genotypes: ChAT-IRES-Cre (Jackson Laboratories stock number 006410), VGLUT2-IRES-Cre (Jackson Laboratories stock number 016963) and GAD2-IRES-Cre (Jackson Laboratories stock number 010802).

For all surgeries, anesthesia was induced with 5% isoflurane and maintained at 1.5%. Mice were placed in a Kopf stereotactic frame upon a feedback-regulated heating pad set to 37°C. Ophthalmic ointment (Lacri-lube, Allergan) was applied to protect the eyes and a depilatory agent (Nair, Church & Dwight) was used to remove hair from the surgical area prior to asepsis. After exposing the skull, a stainless steel headplate (custom fabrication, emachineshop.com) was fixed to the skull with cyanoacrylate and secured with two 000–120 screws (Antrin Miniature Specialties Inc.) in the occipital bone, then cemented in place with dental acrylic.

A craniectomy was drilled at a point 1.0 mm lateral and 0.5 mm anterior of bregma, and a Nanoject (Drummond) was used to inject 300–500 nL of AAV1.Syn. Flex.GCaMP6f.WPRE.SV40 (University of Pennsylvania Vector Core) through a pulled micropipette (Drummond) at a depth of 5.0 mm beneath the cortical surface at a rate of 46 nL per minute. Fifteen minutes after the injection was completed, the pipette was withdrawn and a pin vise was mounted to the stereotactic frame to implant a microendoscope lens 0.5 mm in diameter and 8.2 mm long (Inscopix) to a depth of 5.0 mm through the same craniectomy used for virus injection. The lens was fixed to the skull and headplate with cyanoacrylate glue and dental acrylic darkened with carbon (Sigma). Mice were then injected with 0.1 mg/kg of buprenorphine and 300 mL of saline, returned to their cages, and allowed to recover for 1 week before experiments began. Supplementary analgesia (5 mg/kg meloxicam) was provided 6 h after surgery and daily thereafter if indicated. Targeting of virus injection and lens placement was later confirmed histologically (see below for details). Mice with lenses placed outside the BF were excluded from analysis.

### Endoscopic imaging

Two to four weeks after virus injection, a baseplate for a miniaturized integrated fluorescence microscope (Inscopix: 20 × objective, 1440 × 1080 pixel CMOS sensor) was attached to the headplate with dental acrylic darkened with carbon (Sigma; Ghosh et al., 2011; Ziv et al., 2013). This permitted the microscope to be attached for subsequent imaging sessions while maintaining the optimal orientation and working distance relative to the implanted microendoscope. For imaging experiments, mice were briefly anesthetized with isoflurane and placed under head restraint. The microscope was then attached to its baseplate and the focus adjusted. Imaged neurons were located 50–250 μm below the bottom surface of the lens, with a typical field of view 300 μm across or 0.09 mm^2^. LED power ranged 0.2–0.7 mW. Images were acquired at a rate of 20 frames per second. We attempted to target a separate population of neurons during each imaging session by moving the focal plane by approximately 50 μm between sessions and analyzing only neurons that were well-focused.

### Spontaneous behaviors

Mice were fitted with the integrated fluorescence microscope and then returned to their home cage, which was placed inside a sound-attenuated chamber (Med Associates Inc.). Their spontaneous behaviors were recorded for a period of 25–50 min using a webcam (Logitech) and scored manually as one of five categories. *Sitting* was defined as the absence of any observable movement, *moving* involved movements such as rearing and postural adjustments without locomotion. Movement between different locations within the cage was scored as *running*. *Grooming* included rhythmic movements such as scratching or repeated stroking of the face with the forepaws. Investigation or consumption of food (standard chow supplemented with Hartz brand hamster food including seeds and pellets) was scored as *eating*. Activity of all neuronal types was similar during eating and grooming, so these states were analyzed together.

### Go/no-go auditory discrimination task

Mice were trained to perform a head-fixed go/no-go task using an apparatus described previously (Pinto et al., 2013). Mice were water restricted and received all of their water during performance of the task, but were given supplemental water if body weight dropped below 85% of its pre-training value. All training was performed during the light cycle and at a consistent time of day. Mice were first habituated to gentle handling for approximately 1 week and to then head restraint for 1–2 days. Head restraint was achieved by bolting the headplate to two posts on an aluminum plate (Thorlabs) while the mice lay inside an acrylic tube mounted on the same plate. Once mice were familiar with the apparatus, they were conditioned to lick upon presentation of the Go stimulus (a 2 s, 2 kHz, 65 dB pure tone). Licks were detected with an infrared lickometer. Conditioning was repeated until mice achieved 200 successful trials on two consecutive days; this typically required between 2 and 5 days of training. The ultimate task involved repeated trials, each beginning with a 200 ms flash of light on an LCD screen to signify trial start followed after 1 s by either the Go stimulus or the No-go stimulus (2 s, 8 kHz, 65 dB pure tone), randomly selected with equal probability. The first 0.5 s of stimulus presentation constituted a grace period during which licking was inconsequential. If mice licked during the final 1.5 s of stimulus presentation (response period) in a Go trial, stimulus presentation ended and they were rewarded with ~5 μL of water, followed by a 2 s period for reward consumption and a 3 s inter-trial interval. Failure to lick during the 1.5 s response window was counted as a “Miss”. In a No-go trial, licking during the response period (“False Alarm”, FA) triggered an 8 s timeout period during which no new trial was initiated, followed by the 3 s inter-trial interval. In some experiments, False Alarm errors also incurred punishment in the form of compressed air puffed at the mouse's face for 200 ms (“air puff”). Abstaining from licking during a No-go trial (“Correct Rejection”, CR) earned no reward. Imaging began after mice performed the task with accuracy better than d' of 0.5 for two consecutive days. The behavioral apparatus was controlled using Presentation software (Neurobehavioral Systems). Imaging frames were synchronized with events from the behavioral task by acquiring voltage outputs from the Inscopix imaging system and the Presentation behavior software in a custom Labview script (National Instruments).

### Image processing

Images were spatially down-sampled 8 × using Inscopix Mosaic software. No temporal down-sampling was performed. A subset of frames (approximately 1 per 1000) contained artifacts, these were removed and replaced by duplicating the preceding frame using a custom macro for ImageJ (NIH). Motion correction was then performed with the Image Stabilizer plugin for ImageJ (written by Kang Li). For a subset of recordings, additional motion correction was performed using Advanced Normalization Tools (Brian Avants, http://stnava.github.io/ANTs/).

Regions of interest (ROIs) were identified using an activity map (Ahrens et al., 2012) and then manually selected. In cases where blood vessels were present in the imaging field (8 of 32 mice), fluorescence contributed by out-of-focus neuropil was subtracted using the following equation: *Fsubt(t)* = *Fraw(t) – cf* × *Fnp(t)*, where *Fraw* is the uncorrected fluorescence within an ROI, *Fnp* is the fluorescence within a 20 μm ring surrounding the ROI (presumed to originate from the neuropil), and *cf* is a correction factor calculated as the ratio of the fluorescence in a blood vessel and its surrounding neuropil, with the background value of pixel intensities outside the lens subtracted (Pinto and Dan, 2015). In cases where no blood vessels were present in the imaging field, a constant value of 0.6 was used for *cf*, which closely approximates the mean value of *cf* as calculated above (Pinto and Dan, 2015). Slow bleaching of the calcium indicator was corrected for by low-pass filtering with a 300 s sliding window (Pinto and Dan, 2015).

### Analysis

Analyses were performed on Z-scored ΔF/F, with the session mean fluorescence serving as the denominator. For our analysis of neuronal activity during licking, we first defined licking bouts as consisting of licks separated by an interval of <2 s. Changes in neuronal activity at the onset of licking bouts were calculated as the mean activity during 0.5 s following the first lick in a bout minus a baseline of 1.0–0.5 s preceding the first lick, since activity often began to increase prior to licking onset. Individual behavioral sessions were truncated at the last trial in which mice licked. Changes in activity at the time of trial reward or punishment (outcome) were calculated as the mean of a 1 s period after outcome minus the mean of a 0.2 s pre-outcome baseline period. The shorter pre-outcome period was chosen to avoid contamination by stimulus-related activity. Changes related to other task events were calculated as the mean of 1 s post-event minus the mean of a pre-event baseline of 0.5 s. To calculate latencies of responses to air puffs, we set a threshold of 2 × the standard deviation of a baseline period of 2 s preceding the event and then identified the first time point at which ΔF/F exceeded that threshold for at least 5 consecutive frames (0.25 s). Cells without supra-threshold responses were excluded (5 of 56 ChAT, 46 of 288 GAD, and 26 of 156 VGLUT neurons). For calculating latencies of responses to licking and auditory stimuli, the threshold was set as 3 × the standard deviation of a baseline period. The baseline was set from 2 to 1 s preceding licking to include responses with negative latencies. For auditory stimuli the baseline period extended from 2 to 0.5 s preceding the auditory stimulus to exclude responses to the start cue. We considered only the first licks within a bout that were more than 2 s before or after auditory stimuli. All analyses were performed in MATLAB.

### Statistical tests

Results are reported as mean ± SEM unless specified otherwise. We applied the Lilliefors test to determine normality of data sets and then used either *t*-tests or Mann-Whitney U and Wilcoxon signed rank tests, accordingly. All tests were two-sided unless otherwise specified. For factorial experiments we used either ANOVA or Kruskal-Wallis tests followed by Tukey's Honestly Significant Difference *post-hoc* test for multiple comparisons. Multi-factorial experiments were analyzed using 2-way ANOVA with Tukey's *post-hoc* test.

### Histology

After experiments concluded, mice were deeply anesthetized with isoflurane and transcardially perfused, first with 10 mL room temperature saline and then 10 mL chilled 4% PFA. Acetone was used to dissolve the dental acrylic around the microscope baseplate, microendoscope, and headplate. The brain was then dissected from the skull, immersed in 4% PFA for 12 h, then transferred to a solution of 30% sucrose in PBS for 24 h. Brains were then frozen in OCT (Ted Pella) and stored at −80°C prior to cryosectioning on a Microm HM525 cryostat. Implanted lens positions relative to bregma were estimated using the Paxinos and Franklin mouse brain atlas (Paxinos and Franklin, 2012).

Specificity of GCaMP6f expression was confirmed using immunohistochemistry for cholinergic neurons. Sections of 20 μm thickness were mounted on slides, washed in phosphate buffer (PB), permeabilized with 0.3% Triton (Sigma) and then washed again with PB. Slides were then incubated at room temperature for 2 h with 2% normal donkey serum in phosphate buffer and washed with PB before incubating at 5°C for 36 h with anti-ChAT antibody (AB144P, Millipore, 1:1000 in PB). The slides were again washed in PB, then incubated 2 h at room temperature with secondary antibody (A-21447, Invitrogen, 1:400 in PB). Finally, the slides were washed and coverslipped with Fluoromount-G (Southern Biotech).

Fluorescent *in situ* hybridization was used to detect expression of GAD2 and VGLUT2, using their respective probes, following a protocol described elsewhere (Xu et al., 2015). Briefly, 50 μm sections were sliced with a cryostat, collected in a 12-well plate, fixed with 4% PFA, rinsed with PBS, then incubated with Proteinase K buffer. Sections were incubated for 1 h with hybridization buffer, then probes synthesized using published primers (Allen Institute for Brain Science, brain-map.org) were added and the sections incubated at 60°C for 20 h. Labeling was performed with anti-DIG (1093274, Roche, 1:1000 in PB) and Fast Red TR/Naphthol AS-MX (F4523, Sigma) or anti-GFP (GFP-102, Aves, 1:500 in PB) overnight at 4°C. Images were acquired with a Zeiss LSM 780 confocal microscope and cells were manually counted independently by two investigators in ImageJ using the Cell Counter plugin to obtain a mean number of neurons labeled by GCaMP6f and by their cell type specific marker.

### Results

**Figure 1:**
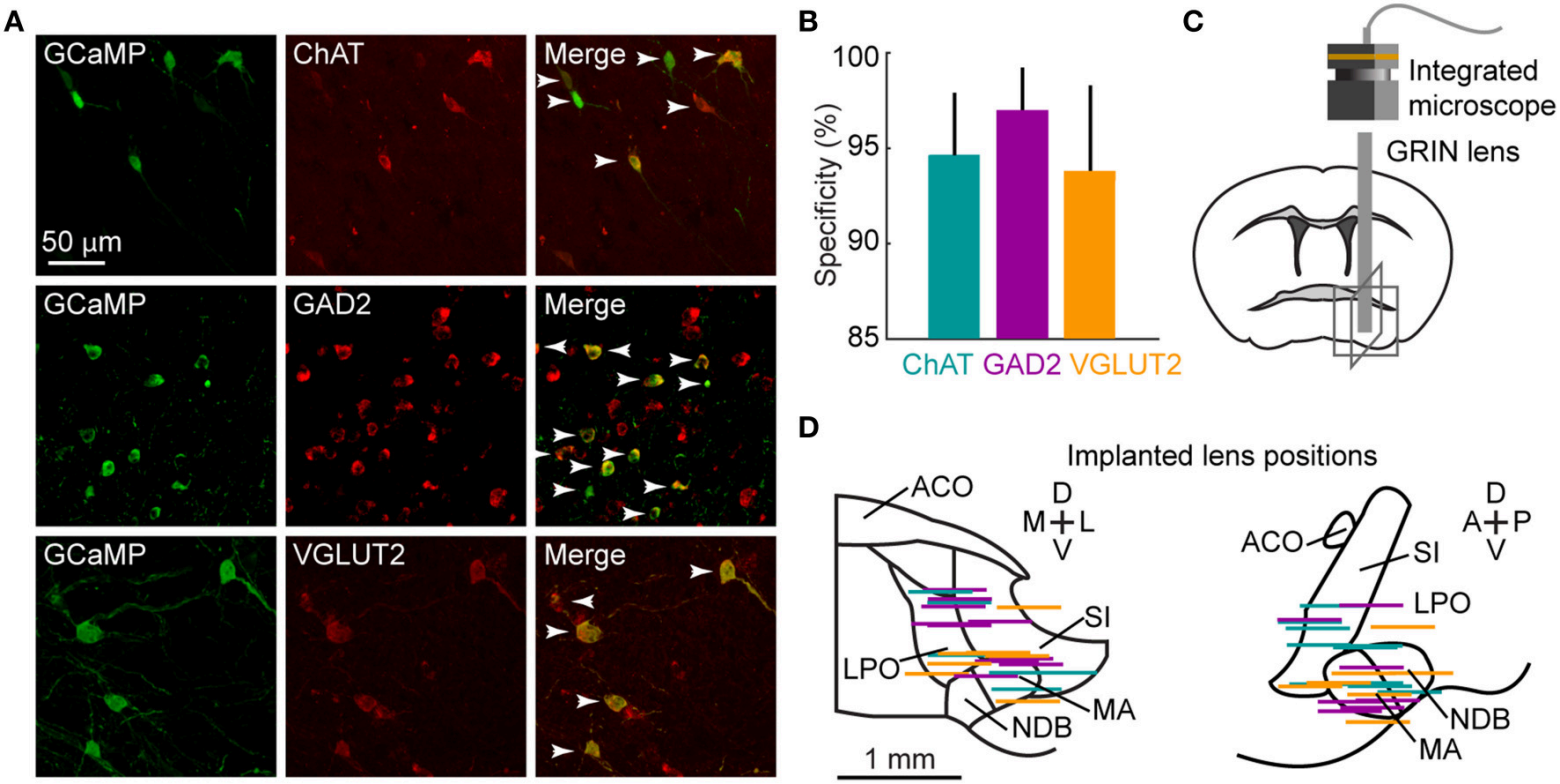
**Microendoscopic calcium imaging in the BF. (A)** Examples of GCaMP6f (green) and cell-type-specific markers (red: ChAT, immunohistochemical staining; GAD2 and VGLUT2, fluorescence *in situ* hybridization) expressed in the respective Cre mice. Arrowheads mark double-labeled neurons. **(B)** Specificity of GCaMP6f expression. 94.4 ± 1.7% of 399 GCaMP6f+ neurons in 5 ChAT-Cre mice; 96.2 ± 1.3% of 879 neurons in 7 GAD2-Cre mice and 93.6 ± 2.4% of 770 neurons in 5 VGLUT2-Cre mice were also labeled by the cell-type-specific marker. Data are presented as mean ± SEM. **(C)** Schematic of microendoscopic imaging in BF. Gray boxes correspond to fields of view in **(D)**. **(D)** Placement of GRIN lenses in the BF, viewed in the coronal (left) and sagittal (right) planes. Each colored line indicates the position of the bottom surface of the lens in one animal. There were no significant differences in implanted lens position between ChAT-, GAD2- and VGLUT2-Cre mice (F_genotype_ = 0.004, *p* = 0.99, 2-way repeated measures (RM) ANOVA, *n* = 9 ChAT, 7 GAD2, 7 VGLUT2 mice). ACO, anterior commissure; LPO, lateral preoptic area; NDB, diagonal band nucleus; MA, magnocellular area; SI, substantia innominata.

To image the activity of each type of BF neurons, we injected adeno-associated virus (AAV) into the BF of ChAT-, GAD2-, and VGLUT2-Cre mice for Cre-dependent expression of the calcium indicator GCaMP6f (Chen et al., 2013). Immunohistochemistry and fluorescence *in situ* hybridization confirmed the high specificity of GCaMP6f expression in all cell types (Figures 1A,B). We then implanted a gradient refractive index (GRIN) lens into the BF, targeting the diagonal band and magnocellular nuclei (NDB and MA, Figures 1C,D). Calcium imaging was performed using a miniaturized integrated fluorescence microscope (Ghosh et al., 2011; Figure 1C).

### BF activity during spontaneous behaviors

**Figure 2:**
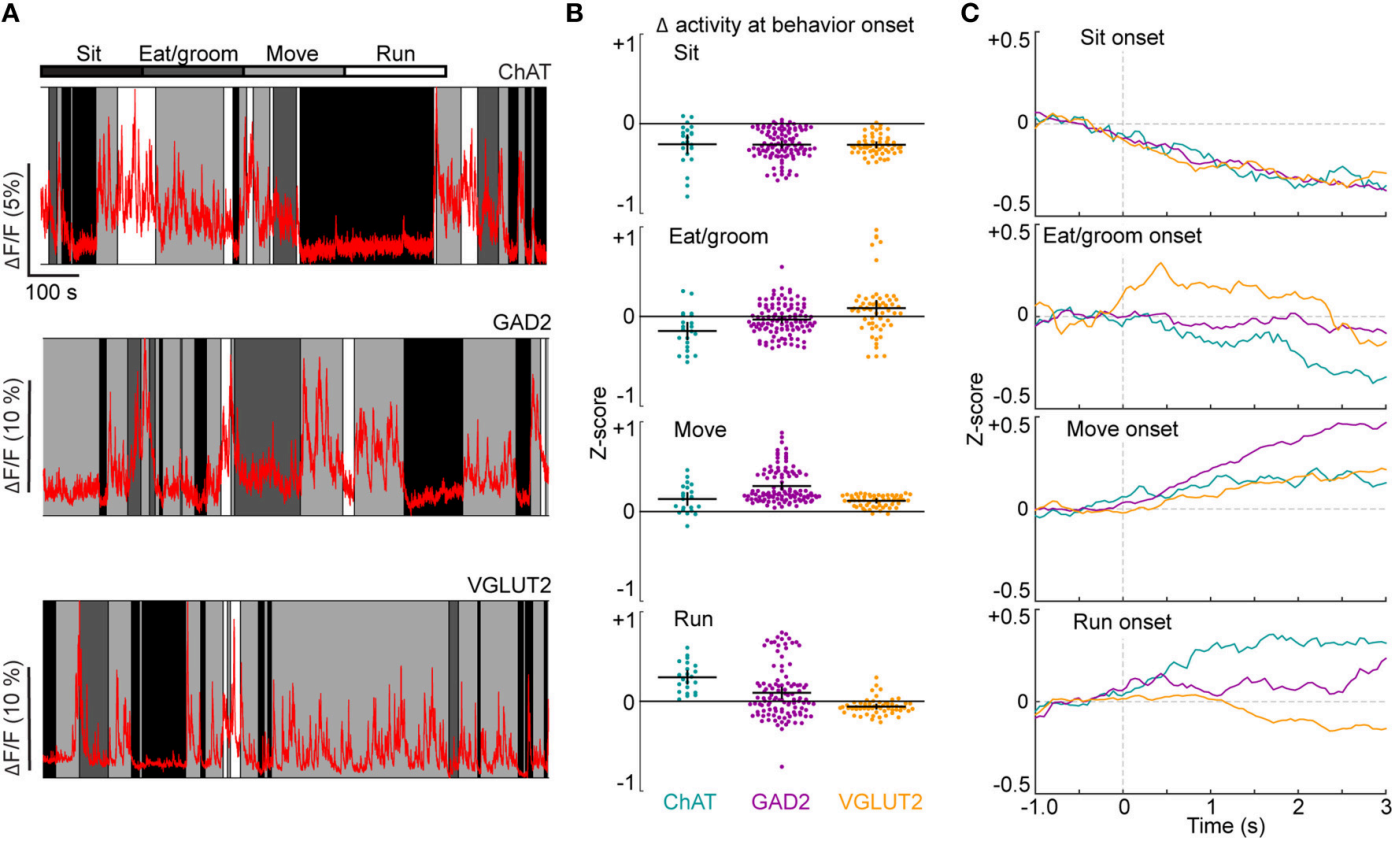
**Imaging neuronal activity during spontaneous behaviors. (A)** Examples of neuronal activity in ChAT-, GAD2-, and VGLUT2-Cre mice. Behaviors (sitting, eating/grooming, moving or running) are indicated by shading. **(B)** Changes in activity in individual ROIs at the onset of each behavior. Black crosses signify mean ± SEM. F_genotype_ = 4.5, p = 0.01; F_behavior_ = 102.7, *p* < 0.01; F_interaction_ = 16.3, *p* < 0.01, 2-way ANOVA, *n* = 22 ChAT, 108 GAD2, 58 VGLUT2 cells. See also Movie S1. **(C)** Mean activity of all ROIs from each cell type, aligned by behavior onset.

We first imaged the activity of cholinergic (ChAT), GABAergic (GAD2), and glutamatergic (VGLUT2) BF neurons as mice engaged in spontaneous behaviors in their home cages (Figure 2A, Movie S1). Based on video recordings, behavior was classified as sitting (total immobility), eating/grooming, moving (including rearing and postural adjustments without running), or running. All cell types were modulated by these spontaneous behaviors, with the lowest level of activity during sitting, moderate activity during eating/grooming and moving, and the highest activity during running (examples in Figure 2A, group F_behavior_ = 379.9, *p* < 0.01, 2-way ANOVA, *n* = 22 ChAT, 108 GAD2, 58 VGLUT2 cells).

We also observed rapid changes in activity at transitions between behaviors, which were quantified as the difference in fluorescence between the 0.5 s period before and 3 s period following the onset of each behavior (Figures 2B,C). The activity of all three cell types decreased at sitting and increased at movement onset (ChAT: *p* = 2.0 × 10^−4^ and 7.8 × 10^−4^, *n* = 22 cells; GAD2: *p* = 5.0 × 10^−19^ and 1.9 × 10^−19^, *n* = 108 cells; VGLUT2: *p* = 3.7 × 10^−11^ and 5.6 × 10^−11^, *n* = 58 cells, Wilcoxon signed rank test). GAD2 neurons were most excited at movement onset (Figure 2B, move vs. sit: *p* < 0.01; move vs. eat/groom: *p* < 0.01; move vs. run: *p* < 0.01; 2-way ANOVA with Tukey's *post-hoc* test, *n* = 108 cells). VGLUT2 cells, but not GAD2 or ChAT cells, became more active at the onset of eating or grooming (*p* = 3.0 × 10^−5^, *n* = 58 cells). In contrast to the diverse changes of GAD2 and VGLUT2 neurons at the onset of running, ChAT cells exhibited a consistent increase in activity (Figure 2B, *p* = 4.0 × 10^−5^, one-sided Wilcoxon signed rank test, *n* = 22 cells, Movie S1). Such a robust activation of BF ChAT neurons is well suited to the function of cholinergic inputs in mediating running-induced gain increases in sensory cortices (Niell and Stryker, 2010; Bennett et al., 2013; Fu et al., 2014; Lee et al., 2014; McGinley et al., 2015).

### BF activity during a go/no-go auditory discrimination task

**Figure 3:**
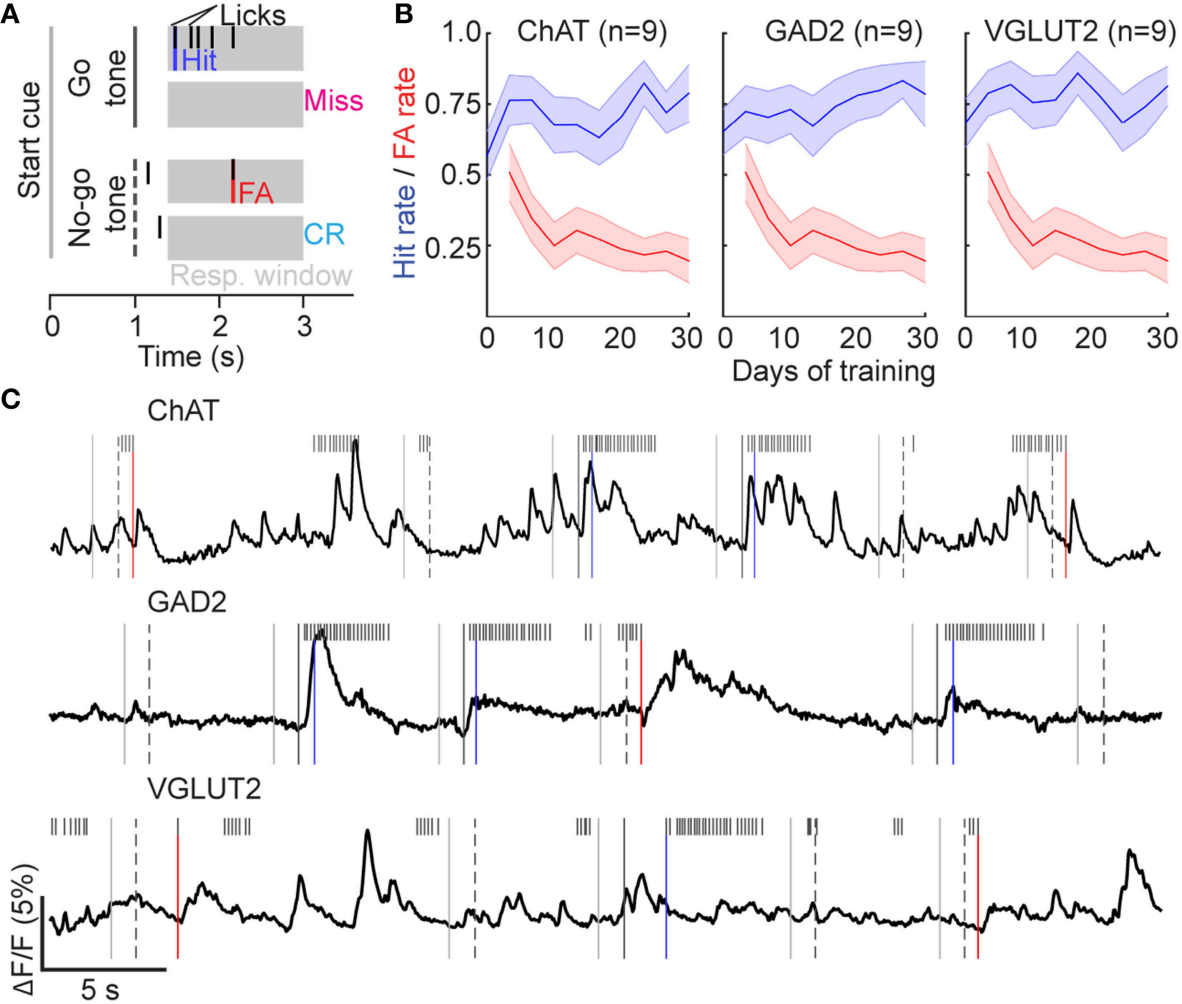
**Go/no-go auditory discrimination task. (A)** Illustration of task structure. **(B)** Hit and False Alarm rates during 3 days of conditioning (Go tone only) followed by 27 days of performing the discrimination task. Line and shading, mean ± SEM for all mice of each genotype. **(C)** Examples of neuronal activity in ChAT-, GAD2-, and VGLUT2-Cre mice performing the discrimination task. Task events are denoted as in **(A)**.

We next characterized BF neuronal activity during a learned task under head-fixed conditions. Mice were trained on a go/no-go auditory discrimination task (Figure 3A, see Experimental Procedures). In each trial, either a “Go” (2 kHz) or a “No-go” (8 kHz) tone was presented. After a grace period of 500 ms (during which licking was inconsequential), licking in response to the Go tone triggered a water reward, and licking to the No-go tone triggered a punishment (air puff and timeout). Mice of all genotypes learned the task with similar time courses (Figure 3B). Calcium imaging showed that the activity of each cell type was modulated by multiple task-related events (Figure 3C).

### Licking-related activity

**Figure 4:**
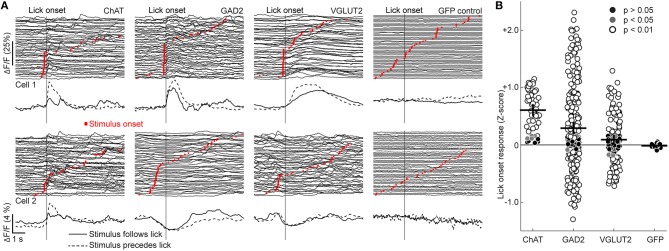
**Lick-related neuronal activity. (A)** Examples of neuronal activity aligned by lick-bout onset (vertical line) and sorted by auditory stimulus presentation time (red dots). The traces below depict mean activity of each cell for lick bouts with the stimulus either preceding (dashed line) or following (solid line) licking onset. **(B)** Changes in ΔF/F (Z-score) of individual ROIs at licking onset. Black crosses signify mean ± SEM. *H*_(3)_ = 43.4, *p* < 0.01, Kruskal-Wallis test, *n* = 51 ChAT, 242 GAD2, 130 VGLUT2 cells. ChAT vs. GAD2: *p* = 3.8 × 10^−6^, ChAT vs. VGLUT2: *p* = 6.5 × 10^−9^, ChAT vs. GFP: *p* = 8.0 × 10^−5^; all other comparisons *p* > 0.05, Tukey's *post-hoc* test.

Visual inspection of the calcium traces revealed robust licking-related activity in many BF cells (Figure 3C). Since most licks were grouped in bouts with short inter-lick intervals within each bout and longer intervals between bouts (Pinto and Dan, 2015), we aligned the activity of each cell by lick-bout onset and averaged across all bouts (see Experimental Procedures). ChAT neurons were consistently excited at lick-bout onset (Figures 4A,B). Interestingly, while some VGLUT2 and GAD2 neurons were also excited (Figure 4A, upper row), others were clearly suppressed (lower row). These licking-related changes in fluorescence signals were not caused by motion artifacts, because in control mice expressing green fluorescent protein (GFP) rather than GCaMP6f, none of the 17 cells we imaged exhibited significant changes in fluorescence at licking onset (Figure 4A, right column, Figure 4B).

Since lick bouts often began shortly after presentation of the auditory stimulus, it is possible that the observed activity at lick-bout onset was in fact triggered by the auditory stimulus. To test this possibility, we analyzed neuronal activity separately for lick bouts either preceded or followed by stimulus presentation (Figure 4A). For each cell type, we found activity changes even in lick bouts without preceding auditory stimuli (Figure 4A, solid traces), demonstrating the existence of genuine licking-related responses in BF neurons. The uniform excitation of ChAT neurons and mixed excitation and suppression of GAD2 and VGLUT2 neurons appear to mirror the activity of these cell types at the onset of running in freely moving mice (Figure 2B, bottom panel). We observed no significant differences between response latencies to licking bouts in the absence of auditory stimuli (Figure S1).

### Sensory-evoked response

**Figure 5:**
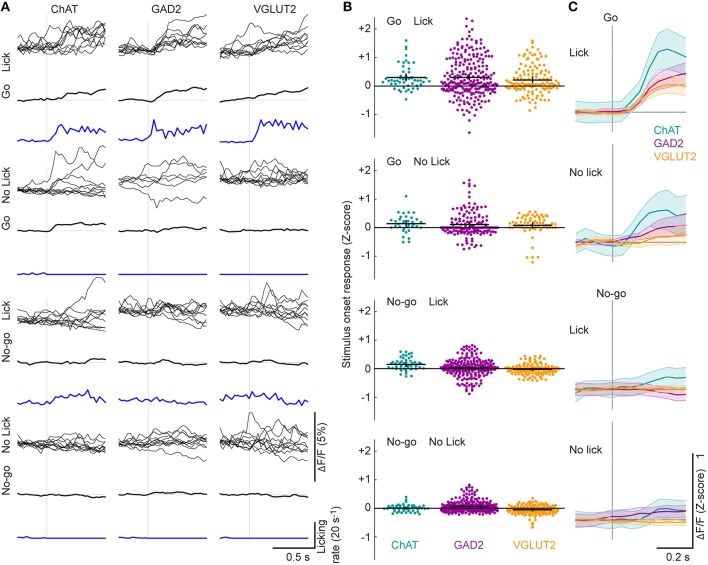
**Stimulus-related neuronal activity. (A)** Examples of single-trial neuronal activity aligned by the Go or No-go stimulus in trials with or without licking during a 1 s period following the stimulus. Mean response across all trials of each type is shown below the single-trial traces of each cell. Licking rate is indicated with a blue line. **(B)** Changes in ΔF/F (Z-score) of individual ROIs at stimulus onset. F_genotype_ = 24.7, *p* < 0.01; F_event_ = 46.6, *p* < 0.01; F_interaction_ = 2.3, *p* = 0.03, 2-way ANOVA, *n* = 51 ChAT, 242 GAD2, 130 VGLUT2 cells. Only ChAT neurons responded more strongly to the Go stimulus than the No-go stimulus in the absence of licking (ChAT: *p* = 4.0 × 10^−4^, *n* = 51; GAD2: *p* = 0.14, *n* = 242; VGLUT2: *p* = 0.53, *n* = 130, Wilcoxon signed rank test). Black crosses, mean ± SEM. **(C)** ΔF/F (Z-score) at stimulus onset, averaged across all ROIs from each cell type. Shading, ± SEM.

To examine whether the Go or No-go sensory stimulus can evoke neuronal responses in the absence of licking, we grouped each type of trials into two sets according to whether the mouse licked within 1 s following the stimulus presentation (Figure 5A). In Go trials with licking, all cell types showed increased activity, some of which is likely associated with licking (Figure 4). In contrast, in the Go trials without licking, only ChAT neurons showed significant increases in activity indicative of sensory-evoked responses (Figure 5B, ChAT: *p* = 1.1 × 10^−5^, *n* = 51; GAD2: *p* = 0.29, *n* = 242; VGLUT2: *p* = 0.35, *n* = 130, one-sided Wilcoxon signed rank test). In trials without licking, the responses to the Go stimulus were significantly larger than those to the No-go stimulus in ChAT neurons (Figures 5B,C, *p* = 4.0 × 10^−4^, *n* = 51, Wilcoxon signed rank test), suggesting that their responses are modulated by task relevance of the sensory cue.

### Punishment activates all BF cell types

**Figure 6:**
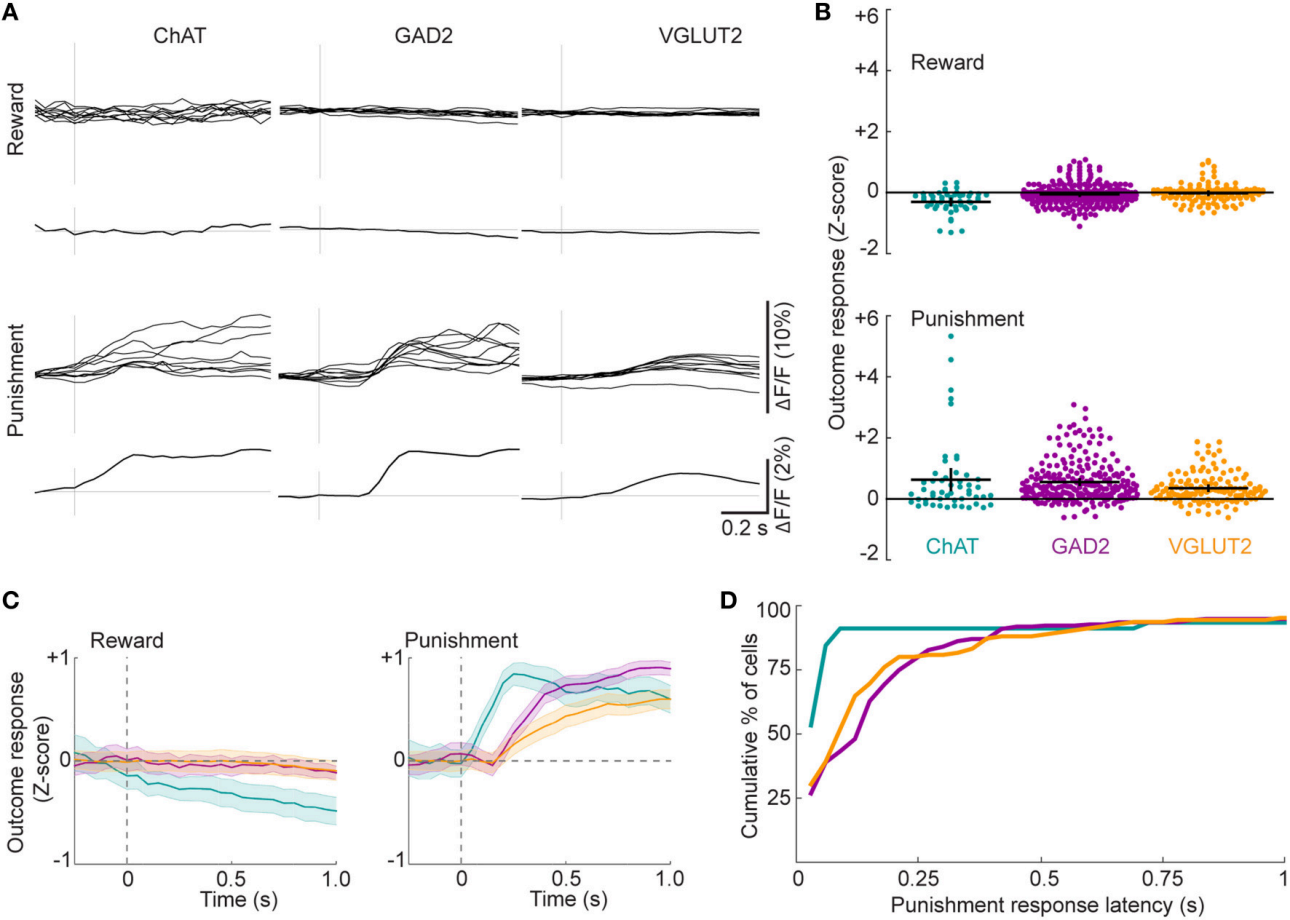
**Reward- and punishment-related neuronal activity. (A)** Examples of single-trial neuronal activity aligned to either reward or punishment. Mean response across all trials of each type is shown below the single-trial traces of each cell. **(B)** Changes in ΔF/F (Z-score) of individual ROIs at the time of reward (top) or punishment (bottom). Black crosses signify mean ± SEM. F_genotype_ = 2.26, *p* = 0.10; F_event_ = 184.16, *p* < 0.01; F_interaction_ = 9.68, *p* < 0.01, 2-way ANOVA, *n* = 51 ChAT, 242 GAD2, 130 VGLUT2 cells. **(C)** ΔF/F (Z-score) at the time of reward (top) or punishment (bottom) averaged across all ROIs from each cell type. Shading, ± SEM. **(D)** Distributions of response latencies for the three cell types after air puff delivery during the go/no-go task.

A recent study showed that BF ChAT neurons respond to punishment and reward (Hangya et al., 2015). However, we rarely detected responses to reward in any cell type, a discrepancy that may be explained by differences in the behavioral task (Figures 6A,B, top, see Discussion). In contrast, punishment evoked clear responses in all cell types (Figures 6A,B, bottom row), which were significantly larger than the reward responses (Figure 6B, ChAT: *p* < 0.01, *n* = 51; GAD2: *p* < 0.01, *n* = 242; VGLUT2: *p* < 0.01, *n* = 130, 2-way ANOVA with Tukey's *post-hoc* test).Punishment-evoked responses exhibited distinct time courses among cell types, with ChAT neurons activated at much shorter latencies than GAD2 and VGLUT2 neurons [Figures 6C,D, *H*_(2)_ = 17.0, *p* < 0.01; ChAT vs. GAD2: *p* < 0.01; ChAT vs. VGLUT2: *p* < 0.01; GAD2 vs. VGLUT2: *p* = 0.99; *n* = 45 ChAT, 231 GAD2, 136 VGLUT2 cells, Kruskal-Wallis test with Tukey's *post-hoc* test], consistent with a previous study based on electrophysiological recordings from cholinergic neurons (Hangya et al., 2015).

**Figure 7:**
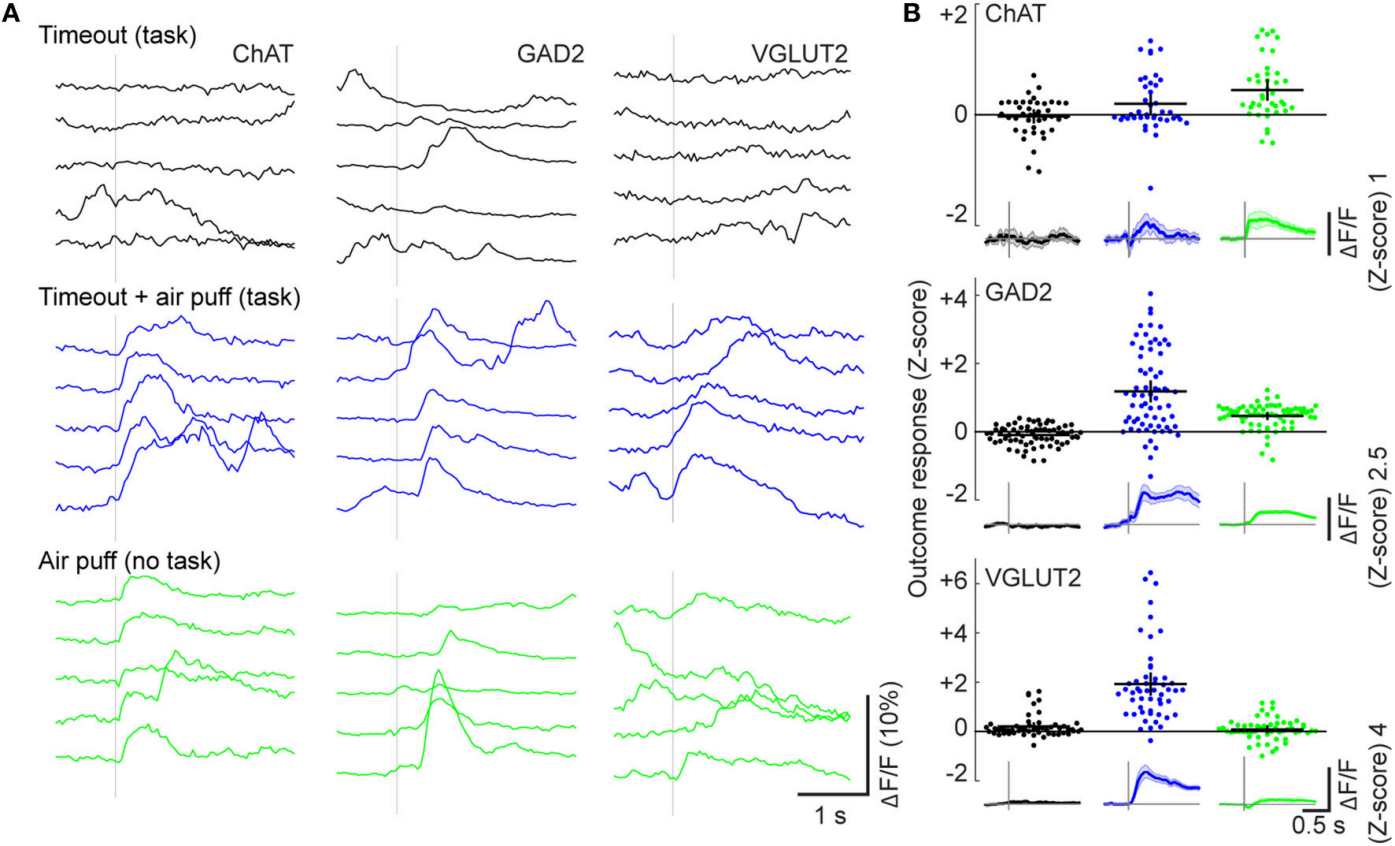
**Dependence of activity on overt punishment and behavioral contingency. (A)** Responses to timeout-only punishment during the task (black, top), timeout and air puff during the task (blue, middle), or random air puff outside of the task (green, bottom). Each panel shows calcium traces from five consecutive trials from an example neuron. **(B)** Changes in ΔF/F (Z-score) in individual ROIs evoked by each form of punishment (color-coded). Black crosses, mean ± SEM. Insets below: ΔF/F traces averaged across all ROIs of each cell type for each form of punishment; Shading, ± SEM.

In our go/no-go task design, punishment consisted of both an air puff and an unrewarded timeout period. To test whether the air puff (overt punishment) is necessary for the BF neuron responses, in a subset of experiments we applied air puff in only 50% of randomly selected False Alarm (FA) trials (timeout was applied in all FA trials, Figure 7). Comparing the mean activity immediately before and after punishment, we found little response to timeout alone (mean change in DF/F Z-score: ChAT: −0.03 ± 0.1, *n* = 41; GAD2: −0.1 ± 0.1, *n* = 67; VGLUT2: 0.2 ± 0.1, *n* = 52), indicating that the mere absence of reward is ineffective in activating BF neurons. For all cell types the peak amplitudes of neuronal responses to air puff were much larger than to timeout alone (Figure 7, ChAT: *p* = 0.03, *n* = 41 ROIs; GAD2: *p* = 2.1 × 10^−11^, *n* = 67; VGLUT2: *p* = 3.5 × 10^−10^, *n* = 52, Wilcoxon signed rank test), indicating that the observed responses in FA trials are primarily attributable to overt punishment.

We then tested whether the BF responses to air puff depend on the behavioral context by delivering air puffs in the absence of any auditory stimuli or contingency on behavior. ChAT cells responded with similar amplitudes to both task-related and randomly delivered air puffs (Figure 7B, *p* = 0.47, *n* = 39, Mann-Whitney U test). In contrast, the response amplitudes of GAD2 and VGLUT2 neurons were significantly higher for task-related than random air puffs (GAD2: *p* = 3.6 × 10^−9^, *n* = 70; VGLUT2: *p* = 1.6 × 10^−15^, *n* = 56), indicating strong modulation by behavioral context.

## Discussion

Using microendoscopes that allow optical access to deep brain structures (Ghosh et al., 2011) and genetically encoded calcium indicators for fluorescence imaging (Chen et al., 2013), we have characterized the activity of all three major BF cell types as mice engaged in innate and learned behaviors. Cholinergic neurons were consistently excited by licking and running behaviors whereas GABAergic and glutamatergic neurons exhibited mixed excitation and suppression. All cell types responded strongly to overt punishment, but cholinergic neurons were activated at much shorter latencies. Thus, whereas all three BF cell types showed robust behavioral modulation on the timescale of subseconds to seconds, the differences between them may support specific functions of each cell type. One caveat of calcium imaging is that calcium transients evoked by action potentials may differ between cell types, which could contribute to the differences we observed between BF cell types in the amplitude and latencies of their responses to various task events.

### Movement-related activity

Previous studies have demonstrated strong cortical activation during running (Niell and Stryker, 2010; Bennett et al., 2013; Polack et al., 2013; Fu et al., 2014; Lee et al., 2014; McGinley et al., 2015). Selective activation of BF ChAT neurons or their axonal projections to the cortex is sufficient to cause cortical activation (Kalmbach et al., 2012; Pinto et al., 2013; Han et al., 2014; Irmak and de Lecea, 2014; Xu et al., 2015), whereas suppression of these neurons or blocking cholinergic transmission in the cortex reduces cortical activation (Pinto et al., 2013; Eggermann et al., 2014; Fu et al., 2014). These studies demonstrated the importance of cholinergic inputs in cortical activation, but whether and how the firing rates of BF ChAT neurons are modulated by running was not addressed. Our finding that cholinergic neurons were strongly excited at the onset of running suggests that the running-related cortical activation is caused, at least in part, by a rapid increase in cholinergic input to the cortex. Interestingly, in head-fixed mice BF ChAT neurons are also activated by licking (Figure 4) and whisking (Eggermann et al., 2014), and electrophysiological recordings in primates showed increased spiking of BF neurons during arm movements (Wilson and Rolls, 1990b). Thus, BF cholinergic neurons may be excited during a broad range of facial and limb movements and as such contribute to cortical activation associated with these movements (Poulet and Petersen, 2008; Eggermann et al., 2014; Castro-Alamancos and Bezdudnaya, 2015).

Unlike cholinergic neurons, GABAergic and glutamatergic BF neurons exhibited a mixture of increased and decreased activity at the onset of running or licking (**Figures 2D, 4**). Since the GABAergic and glutamatergic populations may each contain multiple subtypes of neurons, in future studies it would be interesting to determine whether the different responses at movement onset are associated with distinct projection targets or molecular markers of separate neuronal subtypes. This trend was not apparent during the set of behaviors classified as “move” in this study, perhaps because this category included diverse actions including rearing, manipulation of bedding, and other complex behaviors.

### Responses to reward and punishment

A recent study showed that BF cholinergic neurons respond to both reward and punishment (Hangya et al., 2015), but in our study no consistent response to reward was observed (Figure 6). This may be because cholinergic responses are scaled by uncertainty (Yu and Dayan, 2005; Hangya et al., 2015), and for mice well trained in our task, reward was highly predictable. On the other hand, BF ChAT neurons responded to the Go auditory stimulus (Figure 5), consistent with a previous finding that cholinergic input to the cortex can be activated by reward-predicting sensory cues (Parikh et al., 2007). Interestingly, optogenetic activation of cholinergic neurons increases the percentage of Hit responses in the go/no-go task (Pinto et al., 2013), suggesting that the activity evoked by the Go stimulus (Figure 5) may contribute causally to the behavioral response. Non-cholinergic BF neurons have also been shown to respond to reward, although after the first reward presentation the responses rapidly subsided (Lin and Nicolelis, 2008). This may explain the absence of reward response in glutamatergic and GABAergic neurons imaged in our study.

Unlike reward, overt punishment evoked strong responses in all three BF cell types (Figure 6). The response latency was considerably shorter in cholinergic than GABAergic or glutamatergic neurons. This is consistent with a recent study on cholinergic neurons (Hangya et al., 2015), and it allows these neurons to provide precisely-timed reinforcement signals to their downstream targets (e.g., the cortex) to modulate plasticity and learning (Letzkus et al., 2011; Chubykin et al., 2013; Liu et al., 2015). Responses to punishment have also been observed previously in non-cholinergic BF neurons (Lin and Nicolelis, 2008; Hangya et al., 2015). Our study shows that they are prevalent in both glutamatergic and GABAergic neurons.

Together, our results show that cholinergic, GABAergic, and glutamatergic BF neurons are each modulated by multiple events during innate and learned behaviors, on a time scale of subseconds to seconds. While the three cell types can be excited by some common stimuli (e.g., punishment), albeit with different time courses, they exhibit different activity patterns during other behavioral events (e.g., running or licking). These behavioral modulations of BF activity may allow the three cell types to provide common as well as distinctive modulatory signals to their respective downstream targets to achieve optimal control of behavior.

## Author contributions

TH and YD conceived the work. TH and JB performed the experiments. TH and LP analyzed data. All authors participated in writing the manuscript.

### Conflict of interest statement

The authors declare that the research was conducted in the absence of any commercial or financial relationships that could be construed as a potential conflict of interest.

## Supplementary material

Movie S1 **Microendoscopic calcium imaging of cholinergic neuronal activity in the basal forebrain of a free-moving mouse**. Behavioral video (left) is synchronized with the calcium imaging movie (right) and the ΔF/F trace (right, bottom) for the ROI (indicated by circle) in the basal forebrain of a ChAT-Cre mouse.

Figure S1 **Latencies of neuronal responses to licking and auditory stimuli. (A)** Distributions of response latencies for each cell type following lick bursts in the absence of auditory stimuli. *H*_(2)_ = 4.2, *p* = 0.12, *n* = 44 ChAT, 171 GAD2, 82 VGLUT2 cells with supra-threshold responses. **(B)** Latencies of responses to the Go stimulus in trials with licking. *H*_(2)_ = 1.1, *p* = 0.59, Kruskal-Wallis test, *n* = 43 ChAT, 170 GAD2, 90 VGLUT2 cells. **(C)** Latencies of responses to the Go stimulus in trials without licking. *H*_(2)_ = 9.58, *p* = 8.3 × 10^−3^, Kruskal-Wallis test, *n* = 11 ChAT, 69 GAD2, 27 VGLUT2 cells. ChAT vs. GAD2: *p* = 0.15, ChAT vs. VGLUT2: *p* = 7.8 × 10^−3^, GAD2 vs. VGLUT2: *p* = 9.7 × 10^−2^, Tukey's *post-hoc* test. **(D)** Latencies of responses to the No-go stimulus in trials with licking. *H*_(2)_ = 25.6, *p* = 2.8 × 10^−6^, Kruskal-Wallis test, *n* = 35 ChAT, 192 GAD2, 98 VGLUT2 cells. ChAT vs. GAD2: *p* = 7.5 × 10^−4^, ChAT vs. VGLUT2: *p* = 1.5 × 10^−6^, GAD2 vs. VGLUT2: *p* = 0.03, Tukey's *post-hoc* test. **(E)** Latencies of responses to the No-go stimulus in trials without licking. *H*_(2)_ = 0.7, *p* = 0.70, Kruskal-Wallis test, *n* = 11 ChAT, 126 GAD2, 61 VGLUT2 cells.
