# Supplementary figures and images for: Calcium Imaging of Basal Forebrain Activity during Innate and Learned Behaviors
Source: Front Neural Circuits. 2016 May 9;10:36. doi: 10.3389/fncir.2016.00036 (PMC4863728; doi:10.3389/fncir.2016.00036)

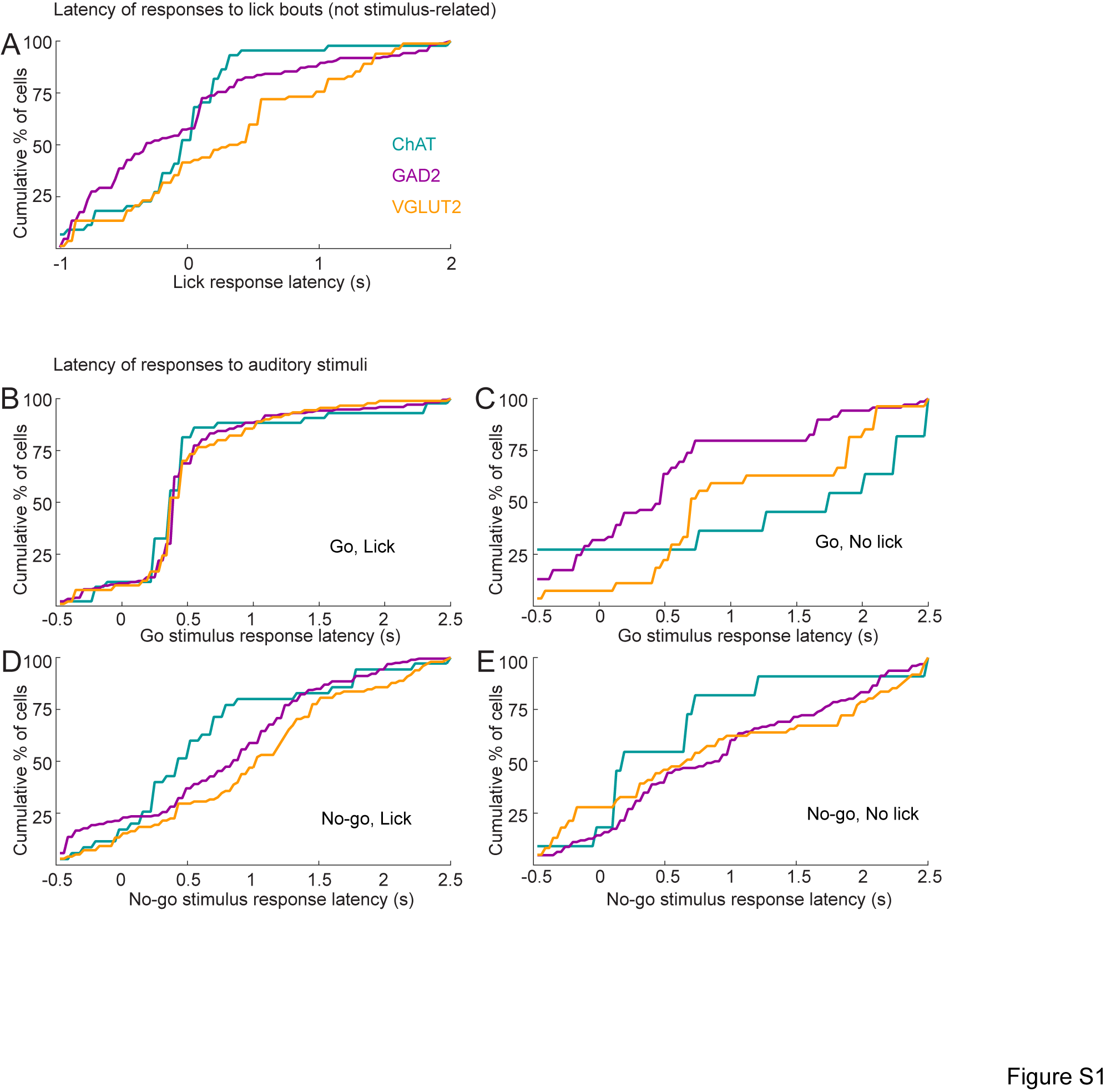

Supplement: Figure S1 — Latencies of neuronal responses to licking and auditory stimuli. (A) Distributions of response latencies for each cell type following lick bursts in the absence of auditory stimuli. H(2) = 4.2, p = 0.12, n = 44 ChAT, 171 GAD2, 82 VGLUT2 cells with supra-threshold responses. (B) Latencies of responses to the Go stimulus in trials with licking. H(2) = 1.1, p = 0.59, Kruskal-Wallis test, n = 43 ChAT, 170 GAD2, 90 VGLUT2 cells. (C) Latencies of responses to the Go stimulus in trials without licking. H(2) = 9.58, p = 8.3 × 10^−3^, Kruskal-Wallis test, n = 11 ChAT, 69 GAD2, 27 VGLUT2 cells. ChAT vs. GAD2: p = 0.15, ChAT vs. VGLUT2: p = 7.8 × 10^−3^, GAD2 vs. VGLUT2: p = 9.7 × 10^−2^, Tukey's post-hoc test. (D) Latencies of responses to the No-go stimulus in trials with licking. H(2) = 25.6, p = 2.8 × 10^−6^, Kruskal-Wallis test, n = 35 ChAT, 192 GAD2, 98 VGLUT2 cells. ChAT vs. GAD2: p = 7.5 × 10^−4^, ChAT vs. VGLUT2: p = 1.5 × 10^−6^, GAD2 vs. VGLUT2: p = 0.03, Tukey's post-hoc test. (E) Latencies of responses to the No-go stimulus in trials without licking. H(2) = 0.7, p = 0.70, Kruskal-Wallis test, n = 11 ChAT, 126 GAD2, 61 VGLUT2 cells. [file Image1.TIF]
